# Supplementary material for: A robust benchmark for detecting low-frequency variants in the HG002 Genome In A Bottle NIST reference material
Source: bioRxiv. 2024 Dec 5:2024.12.02.625685. Preprint. [Version 1] doi: 10.1101/2024.12.02.625685 (PMC11642750; doi:10.1101/2024.12.02.625685)
Supplement: 1 [file NIHPP2024.12.02.625685V1-supplement-1.pdf]

bioRxiv preprint doi: https://doi.org/10.1101/2024.12.02.625685; this version posted December 5, 2024. The copyright holder for this preprint (which was not certified by peer review) is the author/funder. This article is a US Government work. It is not subject to copyright under 17 USC 105 and is also made available for use under a CC0 license.

Supplemental Figures

bioRxiv preprint doi: https://doi.org/10.1101/2024.12.02.625685; this version posted December 5, 2024. The copyright holder for this preprint (which was not certified by peer review) is the author/funder. This article is a US Government work. It is not subject to copyright under 17 USC 105 and is also made available for use under a CC0 license.

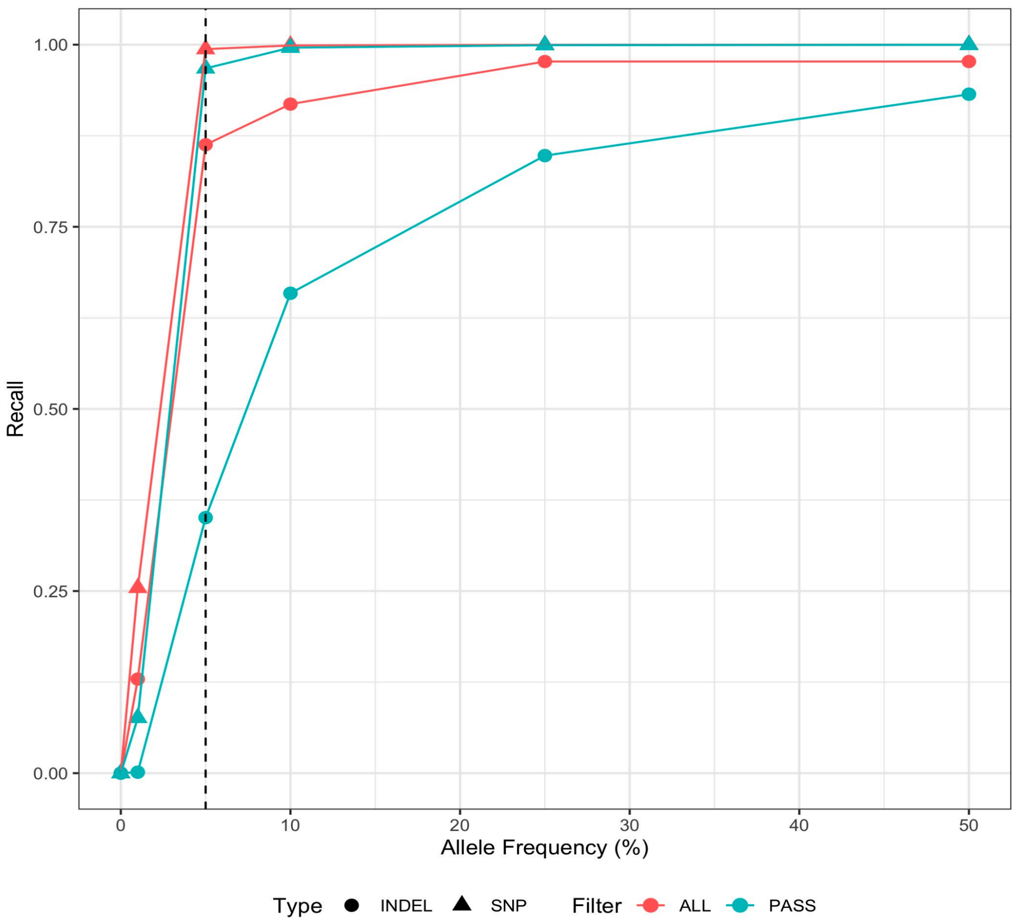

Supplemental Figure 1: Limit of detection (LOD) was established at 5% variant allele fraction (VAF) using Strelka2 callsets from six in-silico mixtures of GIAB reference 300x samples (subset to chromosome 20), HG002 (son) and HG003 (father), as a control. X-axis values indicate the different samples, ranging from 0% AF (HG003 unmixed) to 50% AF (HG002 unmixed). Callsets for each mixture were benchmarked in the precisionFDA app.

bioRxiv preprint doi: https://doi.org/10.1101/2024.12.02.625685; this version posted December 5, 2024. The copyright holder for this preprint (which was not certified by peer review) is the author/funder. This article is a US Government work. It is not subject to copyright under 17 USC 105 and is also made available for use under a CC0 license.

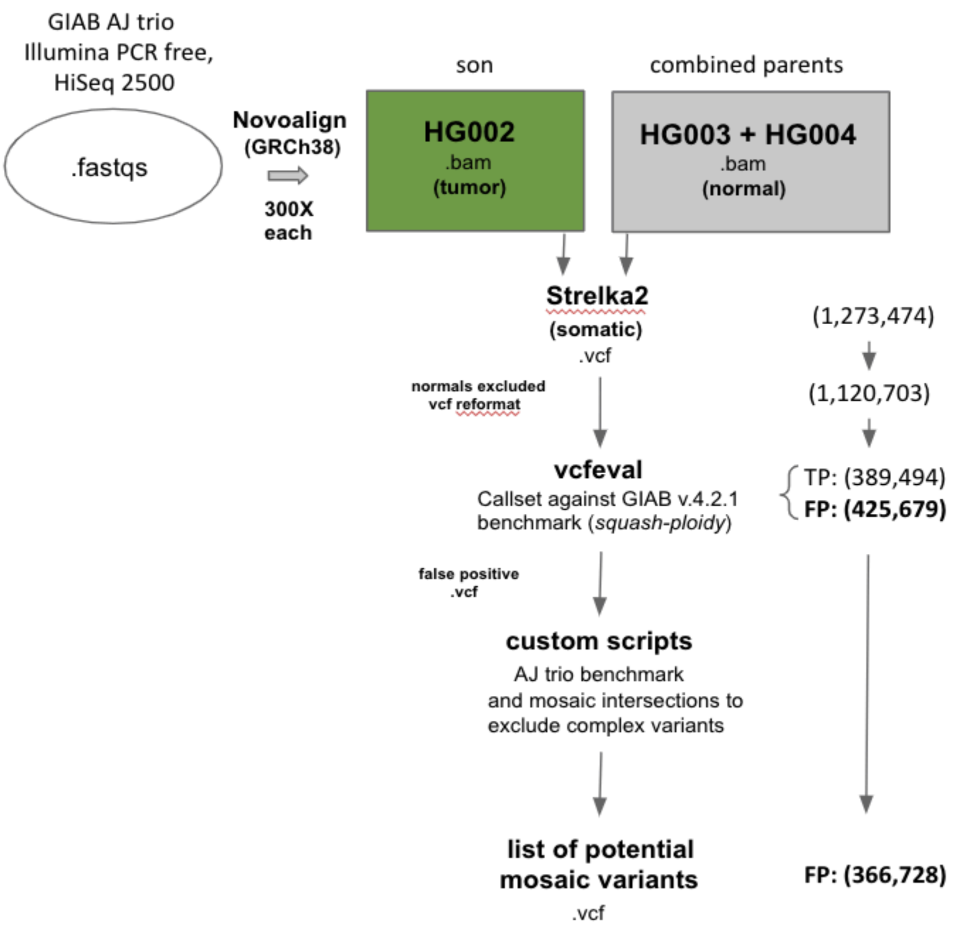

Supplemental Figure 2: Variant counts from the Strelka2 tumor-normal run with the GIAB AJ trio, benchmarking, and filtering steps to generate a list of potential mosaic variants (366,728 vcfeval false positives) for database creation.

bioRxiv preprint doi: https://doi.org/10.1101/2024.12.02.625685; this version posted December 5, 2024. The copyright holder for this preprint (which was not certified by peer review) is the author/funder. This article is a US Government work. It is not subject to copyright under 17 USC 105 and is also made available for use under a CC0 license.

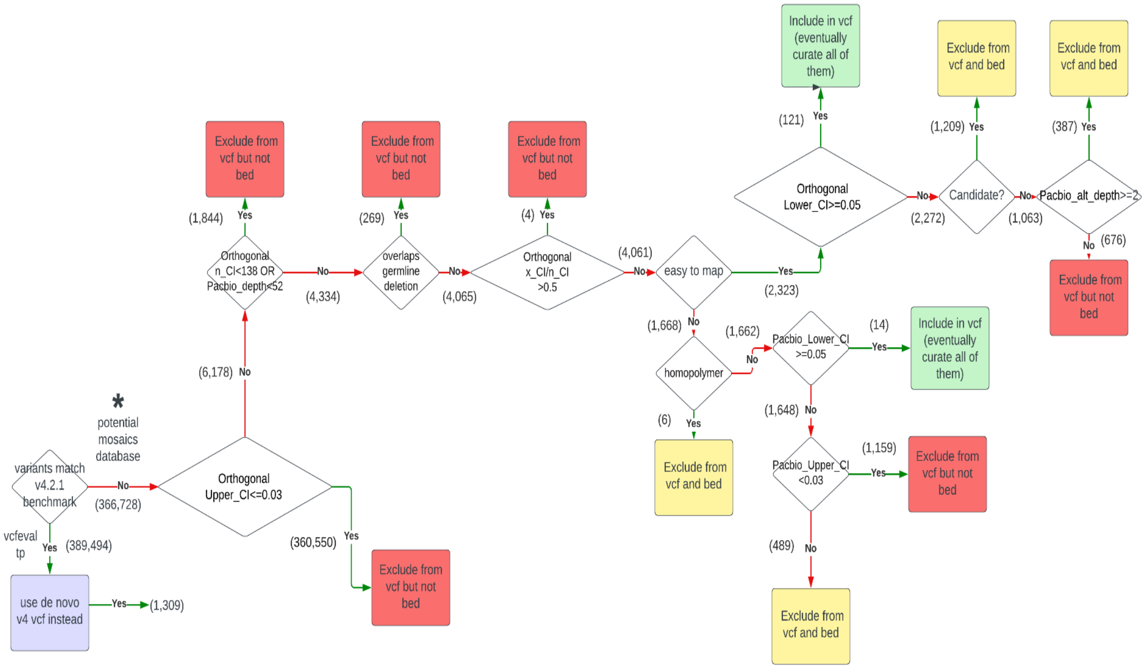

Supplemental Figure 3: Mosaic benchmark decision tree for filtering potential mosaic variants to produce a list for manual curation. A series of heuristics were applied to the potential mosaic database (* starting at bottom left) using combined orthogonal CI thresholds and other attributes for candidate set determination. The tree resulted in three groups of variants: green boxes indicate variants to curate, yellow represents variants excluded from benchmark VCF and BED, and red variants excluded from benchmark VCF but not BED. Values in parentheses are variant counts for each step of the decision tree.

bioRxiv preprint doi: https://doi.org/10.1101/2024.12.02.625685; this version posted December 5, 2024. The copyright holder for this preprint (which was not certified by peer review) is the author/funder. This article is a US Government work. It is not subject to copyright under 17 USC 105 and is also made available for use under a CC0 license.

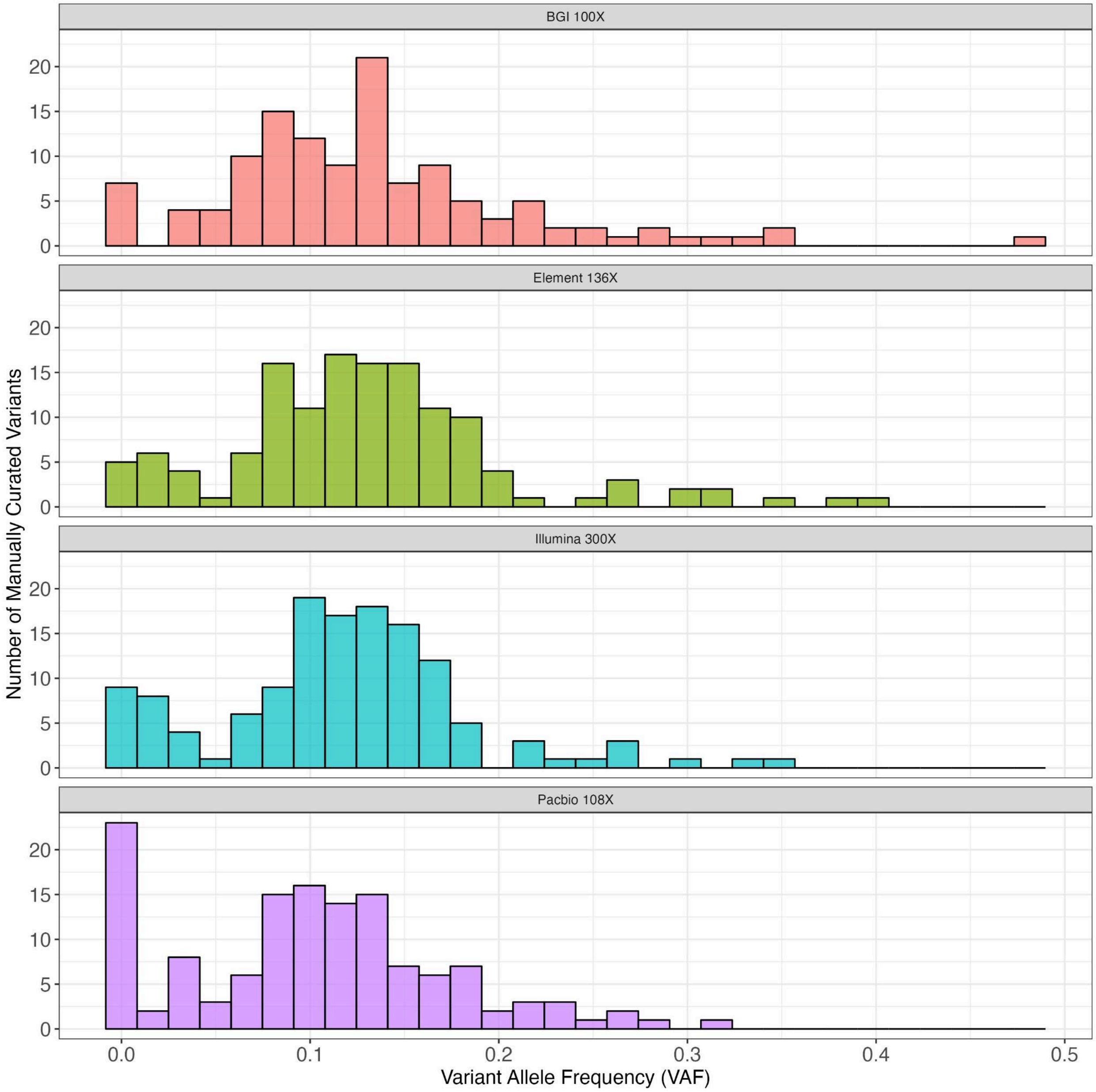

Supplemental Figure 4: VAF distributions for all **manually curated variants** (135) in high-coverage Illumina (300x) and orthogonal (BGI - 100x, Element - 136x, PacBio HiFi - 106x) datasets generated from NIST HG002 reference material.

bioRxiv preprint doi: https://doi.org/10.1101/2024.12.02.625685; this version posted December 5, 2024. The copyright holder for this preprint (which was not certified by peer review) is the author/funder. This article is a US Government work. It is not subject to copyright under 17 USC 105 and is also made available for use under a CC0 license.

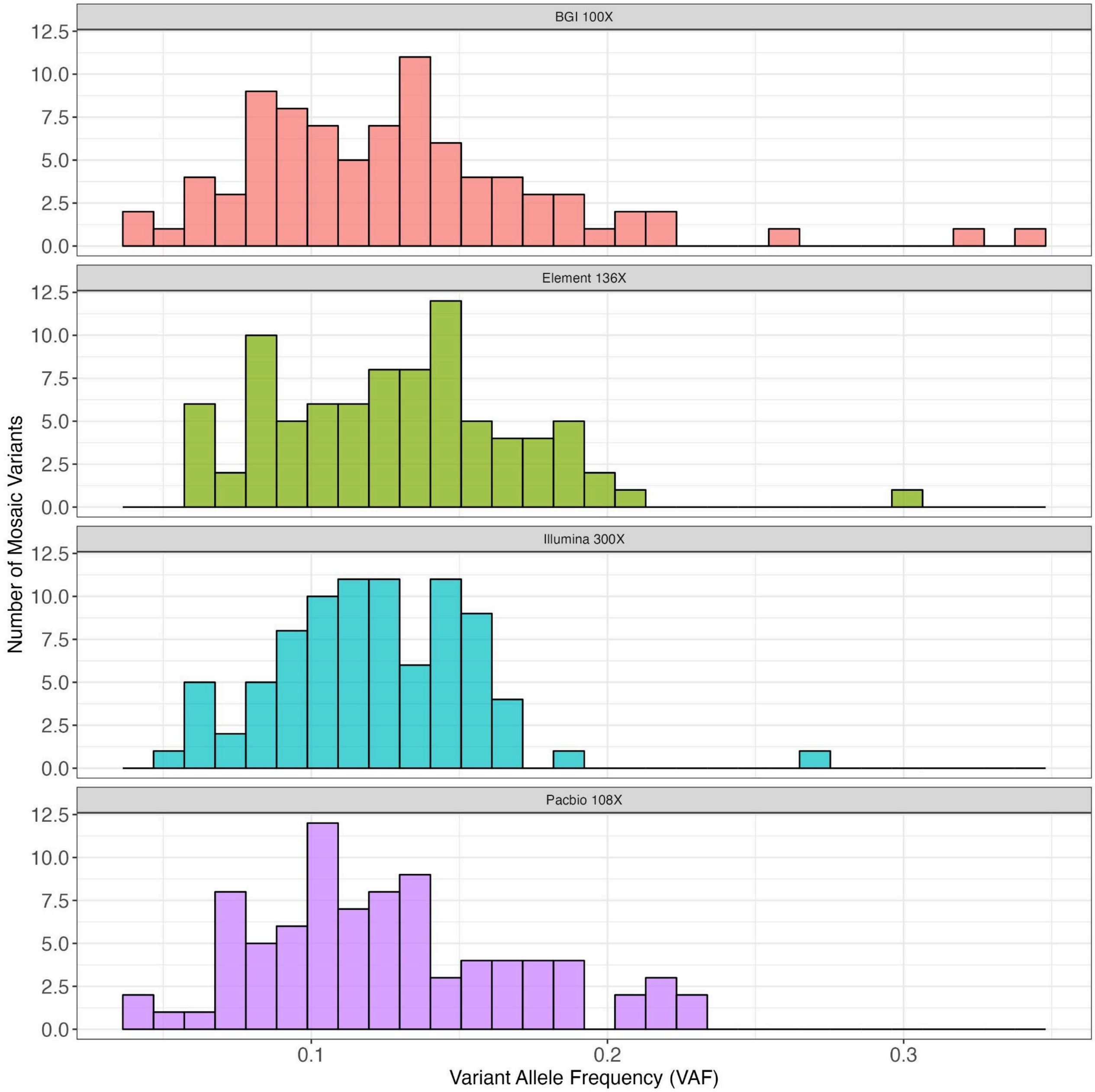

Supplemental Figure 5: VAF distributions for the 85 HG002 **mosaic benchmark variants** in high-coverage Illumina (300x) and orthogonal (BGI - 100x, Element - 136x, PacBio HiFi - 106x) datasets generated from NIST HG002 reference material.

bioRxiv preprint doi: https://doi.org/10.1101/2024.12.02.625685; this version posted December 5, 2024. The copyright holder for this preprint (which was not certified by peer review) is the author/funder. This article is a US Government work. It is not subject to copyright under 17 USC 105 and is also made available for use under a CC0 license.

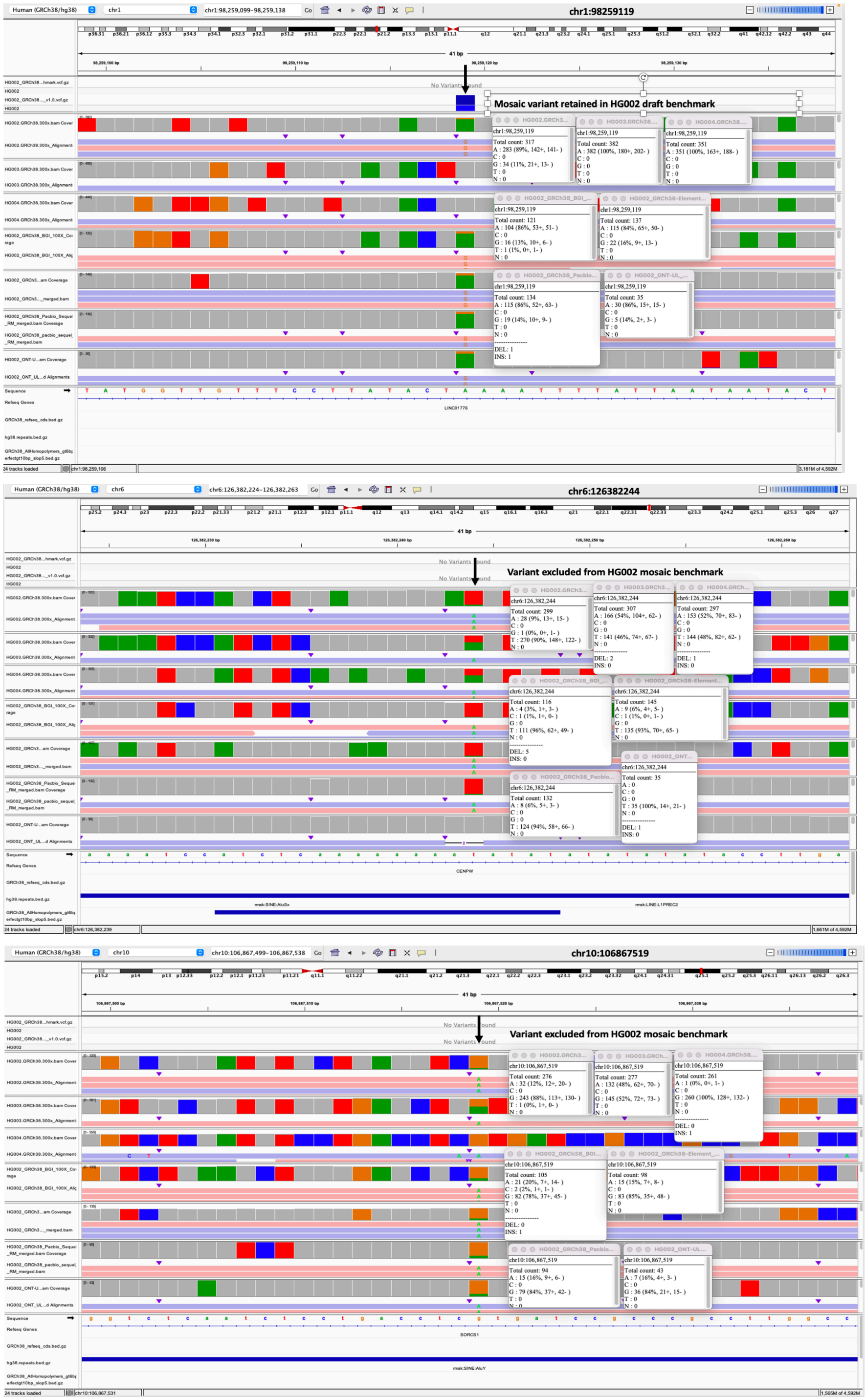

Supplemental Figure 6: IGV profiles of variants (A) included in and (B,C) excluded from the HG002 mosaic benchmark after manual curation. Profile A depicts an HG002-specific candidate mosaic SNV (chr1:98259119 - not detected in parents), while B and C show a putative and candidate SNV (chr6:126382244 and chr10:106867519). The first three data tracks are the HG002 (son), HG003 (mother), and HG004 (father) 300X coverage Illumina data. The following tracks are for HG002 BGIseq dataset, Element, PacBio Sequel, and ONT-UL datasets.
